# Supplementary material for: Chromothripsis during telomere crisis is independent of NHEJ, and consistent with a replicative origin
Source: Genome Res. 2019 May;29(5):737–49. doi: 10.1101/gr.240705.118 (PMC6499312; doi:10.1101/gr.240705.118)
Supplement: Supplemental Material [file supp_gr.240705.118_Supplemental_file_1.zip › contigs/annotated_contigs/DB51/contig.2.DB51_length_716_mean_cov_7.87430167598.docx]

**DB51_length_716_mean_cov_7.87430167598**

```
GAACTTATATTTGACAGAAGTTCATTACATTTCAATTTAAGTATGTTACGATTTTTATCCTTGGGTAACTTGGTGTCTATGTTAGTGGA
 >chr13:90244634-90245012 - E=1e-215
GGAGGGAGGTTCAAAAACAATAAATGATGTAAGAGTTGAGAGTTCAACTCAGGTTATATTATATGATATAAAATAACACCGATGTGAAG

AATGGGAGGTAAAACTTTAAAAAAAGTAAAATGTAGAAAACTTAAAGAAATTGAGGCATTGGCTGGATGTGGTGGCTCACACTTGTAAT

CCCAACACTTTGGGAGGCTGAGGTGGGCGGATCATGAGGTCCGGAGATCGAGACCATACTGGCTAATGTGGTGAAACCTGGTCTCTACT

AAAAATACAAAAAAAATTAG|CT|AACCACTAAGATTTATAGGGATAAGGGCCCACAGTATGGTGGTTTGGTATACAATCTACCATACA
                     >chr13:90232528-90232868 - E=9e-193
TTTGTATGTTCATTTGAATTCTTAATAACACAGGGTTGCTGAAATTTTGGCAATTTCTATTTCCATATATTTTCTATAATGGTTTGTCA

AATGGGAAACATAAAGTTAAAAATGACATTTAAATTACTAGGGAAGACATTCCTAATTCATCTTATCCACTTGACGCTGAGACTTGGAA

CTTCTGTTCTTGTGTACATTTAGTGACATATTTGTGACATATTAAATTTTTTTTAATGTTCTCCAACATGAATATTCTTCTGTTATTCT

GCACAG
```
